# A method to investigate radial glia cell behavior using two-photon time-lapse microscopy in an *ex vivo* model of spinal cord development

**DOI:** 10.3389/fnana.2014.00022

**Published:** 2014-04-10

**Authors:** Janelle M. P. Pakan, Kieran W. McDermott

**Affiliations:** Department of Anatomy and Neuroscience, University College CorkCork, Ireland

**Keywords:** progenitor cell, electroporation, organotypic slice culture, brain lipid binding protein (BLBP), spinal cord

## Abstract

The mammalian central nervous system (CNS) develops from multipotent progenitor cells, which proliferate and differentiate into the various cell types of the brain and spinal cord. Despite the wealth of knowledge from progenitor cell culture studies, there is a significant lack of understanding regarding dynamic progenitor cell behavior over the course of development. This is in part due to shortcomings in the techniques available to study these processes in living tissues as they are occurring. In order to investigate cell behavior under physiologically relevant conditions we established an *ex vivo* model of the developing rat spinal cord. This method allows us to directly observe specific populations of cells *ex vivo* in real time and over extended developmental periods as they undergo proliferation, migration, and differentiation in the CNS. Previous investigations of progenitor cell behavior have been limited in either spatial or temporal resolution (or both) due to the necessity of preserving tissue viability and avoiding phototoxic effects of fluorescent imaging. The method described here overcomes these obstacles. Using two-photon and confocal microscopy and transfected organotypic spinal cord slice cultures we have undertaken detailed imaging of a unique population of neural progenitors, radial glial cells. This method uniquely enables analysis of large populations as well as individual cells; ultimately resulting in a 4D dataset of progenitor cell behavior for up to 7 days during embryonic development. This approach can be adapted to study a variety of cell populations at different stages of development using appropriate promoter driven fluorescent protein expression. The ability to control the tissue micro-environment makes this *ex vivo* method a powerful tool to elucidate the underlying molecular mechanisms regulating cell behavior during embryonic development.

## Introduction

Living cells in the central nervous system (CNS) are highly dynamic, especially during embryonic stages, and a full analysis of developmental processes can only be achieved using techniques that directly monitor changes over time (Lichtman and Fraser, 2001). Live-cell microscopy provides the opportunity to study these dynamic developmental events at both the neural network level and single-cell level. However, such experimental procedures present several challenges (Dailey et al., 2011) and a balance between experimental control and physiological relevance of the preparation must be found.

Traditionally, the properties of stem cells (such as factors affecting self-renewal and determinants of multipotency and lineage) have been studied *in vitro*, often using neurosphere assays (Gage, 2000; Temple, 2001; Costa et al., 2011). While these studies provide many advantages, cell culture conditions and the absence of the appropriate micro-environment may significantly alter progenitor cell properties (Gabay et al., 2003; Czekaj et al., 2012). These issues can be addressed using *in vivo* investigations (Lichtman and Fraser, 2001), however, to date, few *in vivo* studies directly imaging the adult spinal cord have been performed in mammals (Misgeld et al., 2007; Davalos and Akassoglou, 2012; Steffens et al., 2012; for review see Johannssen and Helmchen, 2013; Laskowski and Bradke, 2013) and investigating developmental cell behaviors in the embryonic mammalian spinal cord presents even more challenges. This is largely due to technical complications, such as the inaccessibility of embryos for imaging, difficulties in maintaining the health of embryos *ex utero* (Udan and Dickinson, 2010; Piliszek et al., 2011) and poor experimental control of variables. Although technological innovations are rapidly advancing *in vivo* research, there is currently a need for model systems with greater pharmacological and molecular experimental control than *in vivo* systems presently offer, while still providing physiological relevance.

*Ex vivo* preparations, such as tissue explants and CNS tissue slices, offer such model systems (Cho et al., 2007). While cortical and hippocampal slice models have been well established for some time (Stoppini et al., 1991; Elias and Kriegstein, 2007; Fuller and Dailey, 2007; Gertz et al., 2014), comparatively few studies have examined developmental processes in the spinal cord using *ex-vivo* preparations. Many of the interesting models that have been developed are acute preparations that are often very useful for electrophysiological and neuroanatomical tracing studies (Hanson and Landmesser, 2003; Szokol and Perreault, 2009; Perreault and Glover, 2013), but not necessarily for extended live-cell imaging; a vital component when examining dynamic developmental processes.

Tubby et al. (2013) recently established a slice culture protocol to examine motor neuron development in embryonic chick spinal cord. Using this method they found conserved transcription factor domains, normal motor neuron survival rates and the migration of motor neurons to appropriate positions in the spinal cord after 24 h in culture. These findings are important to establish spinal cord slice culture as a viable model system to investigate normal developmental processes, however, this study did not directly image cell behavior over the course of the experiments. Previous protocols have been established to examine embryonic chick spinal cord segments (Das et al., 2012) and peripheral nerve outgrowth in organotypic spinal cord slices from mouse embryos (Brachmann and Tucker, 2011), both using widefield microscopy. While these studies are very informative, imaging living tissues using widefield microscopy presents a number of disadvantages, namely increased levels of phototoxicity (limiting the extent of the imaging period), and decreased depth penetration and resolution. Therefore, while the introduction of methods to examine the neuroanatomical and physiological properties of the spinal cord using *ex vivo* models is encouraging, the ability to follow specific cell populations during spinal cord development over extended periods of time as well as examine developmental processes on a cellular level remains elusive.

With advances in live-cell microscopy and genetic labeling of distinct cell types in the developing brain (Schmid et al., 2006; Higginbotham et al., 2011; Nowotschin and Hadjantonakis, 2014), the analysis of fundamental processes of CNS formation has become possible. Here we report the development of an *ex vivo* method that allows us to directly record the complex developmental behavior of identified progenitor populations and individual progenitor cells. This is achieved using organotypic spinal cord slice cultures, transfection techniques to isolate specific progenitor cell populations [brain lipid binding protein (BLBP) expressing radial glial cells; Feng et al., 1994; Barry et al., 2014] and two-photon microscopy to produce high resolution, extended time-lapse imaging. With this enhanced ability to investigate progenitor cell behavior during development, we are gaining insight into how a simple tube of undifferentiated neuroepithelial cells will eventually develop into the complex network of the adult spinal cord.

## Materials and methods

All procedures were performed under a license issued by the Irish Government Department of Health and in accordance with the EU Directive 2010/63/EU. Embryonic tissue from time mated Sprague-Dawley rats (Biological Services Unit, University College Cork, Ireland) at embryonic day (E)12-E16 was used in this study.

### Organotypic slice culture and transfections

Pregnant rats were anesthetized with isoflurane followed by decapitation, embryos were collected by laparotomy and placed into ice cold sterile Hank's balanced saline solution (HBSS; Sigma Aldrich, UK). In young embryos (E12–E14) the whole embryo was embedded in 4% low melting agarose [in phosphate buffered saline (PBS) with 5 mg/ml glucose; Sigma Aldrich, UK]. For older ages (E16 and above) it is useful to make a gross dissection of the spinal column region before embedding in agarose. Using these methods preserves the surrounding non-neural tissue elements, resulting in a preparation that more closely resembles the natural tissue environment. The agarose block containing the tissue was trimmed so that the tissue was surrounded by about 2 mm of agarose on all sides. Transverse sections (400 μm) were sliced in ice cold L15 dissecting media (Sigma Aldrich, UK) using a vibratome (VT1200, Leica Microsystems). An important criterion for tissue viability was the use of a high quality vibratome for slicing and, optimally, using a slow cutting speed (0.03 mm/s), high amplitude of blade vibration (1.5 mm) and slice thickness between 300–400 μm.

Using the interface method (Stoppini et al., 1991) slices were positioned on Millicell inserts (PICM0RG50; Millipore, Germany) or Alvatex 3D culture inserts (STP004; Reinnervate, UK) placing 3–4 slices onto each membrane and placing the membrane in 6-well plates containing 1 ml of culture medium per well [phenol red free DAddulbecco's Modified Eagle's Medium (DMEM; Gibco, Ireland) containing 25 mM HEPES, supplemented with HBSS (25% v/v), D-glucose (4.5 mg/ml), penicillin (100 U/ml), streptomycin (100 U/ml), L-glutamine (2 mM), and 10% heat inactivated fetal calf serum].

**Figure 1:**
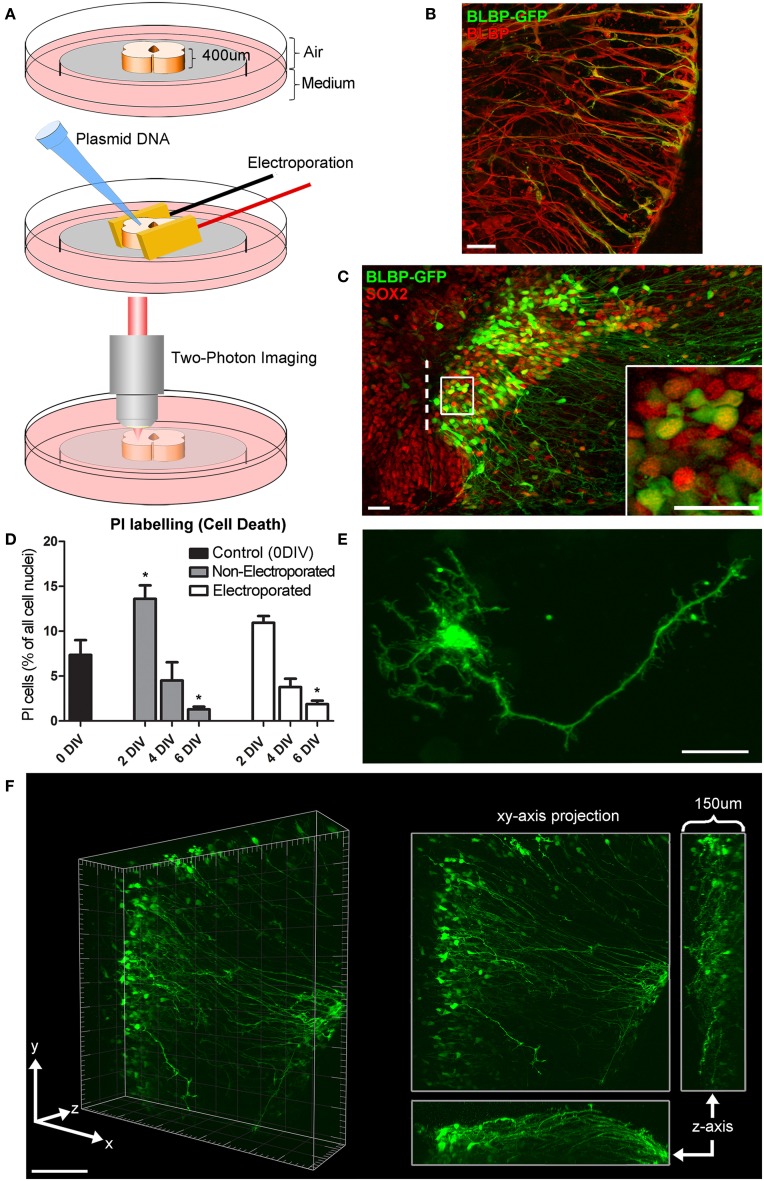
**Organotypic spinal cord slice culture and transfection with a BLBP-GFP plasmid. (A)** 400 μm thick spinal cord slices are electroporated and imaged in a 35 mm dish using two-photon microscopy. For simplicity, only the spinal cord itself is shown in this schematic, although the surrounding non-neural tissues in these slices is also preserved. **(B)** Confocal image showing co-labeling of radial glial cell fibers after transfection with BLBP-plasmid (green) and BLBP immunohistochemistry (red). **(C)** Confocal image of transfected spinal cord slice showing GFP expression (green) and SOX2 immunohistochemistry (red), dashed line represents the lumen (midline); the percentage of progenitor cells transfected (transfection efficiency) is calculated based on the number of SOX2 positive cells also expressing GFP on the transfected side (see inset for example). **(D)** Tissue viability was assessed at 0 (control) 2, 4, and 6 DIV in electroporated and non-electroporated slices; PI labeling was used as a marker for cell death and expressed as a percentage of the total number of cells per slice. Percentage of PI labeling increased at 2DIV (^*^p = 0.0173 in non-electroporated slices) and decreased after 6 DIV (^*^p = 0.0153 in non-electroporated; ^*^p = 0.023 in electroporated slices) compared with 0 DIV controls. There were no significant differences in PI labeling between non-electroporated and transfected slices at any timepoint. **(E)** Two-photon projected image of GFP-expressing cell showing the high spatial resolution achievable. **(F)** Two-photon z-stack showing GFP-expressing radial glial cells in a spinal cord slice after electroporation at E14; left panels shows a 3D tissue block of approximately 500 × 500 × 150 μm at a single timepoint; right panel shows maximum intensity projection in the x,y, and z planes. In all photomicrographs dorsal is oriented toward the top, ventricular zone toward the left, and the pial surface is toward the right of the image. Scale bar in **(B,C,E)** = 25 μm; **(F)** = 150 μm.

After slices had recovered for 1 h, slice electroporation was carried out directly on culture inserts (Figure 1A) using a pair of gold-plated Genepaddles™ and a BTX ECM 830 Square Wave Electroporator (Harvard Apparatus, UK). Plasmid DNA (BLBP-GFP; where GFP expression is driven by a BLBP promotor specific to radial glial cells; kindly gifted by Dr. Eva S. Anton, University of North Carolina; Schmid et al., 2006) was applied directly to each tissue slice, in the region of the lumen, immediately before electroporation (2 μl of 1.0 μg/μl DNA). The electroporation parameters used were optimized for our preparations and according to previously studies (Murphy and Messer, 2001); Genepaddles™ were placed on either side of the spinal cord slice, in order to avoid any damage to the spinal cord itself, and five pulses (50 ms duration pulse, 950 ms interval) were applied at a voltage between 50–70 V depending on the age of the embryonic tissue, older ages requiring higher voltages for optimal transfection. After electroporation, plates containing tissue slices were placed in a humidified incubator with 5% CO2 at 37°C until imaging commenced.

### Cell viability in organotypic slice cultures

Tissue viability was determined using the fluorescent indicator propidium iodide (PI), which is a marker for the loss of plasma membrane integrity and therefore cell death (Noraberg et al., 1999). Slices at 0 (control), 2, 4, and 6 days *in vitro* (DIV) were immersed in a PI solution (2 μg/ml in culture media), incubated for 15 min, rinsed with PBS, fixed with 4% paraformaldehyde (PFA), counterstained with bisbenzimide (1:3000; Sigma-Aldrich, UK) for 5 min, rinsed again in PBS, mounted using Fluoromount-G (eBiosciences, Ireland) and imaged using confocal microscopy (Olympus FV1000, Germany). Cell death was calculated as the percentage of PI labeled cells to total number of cells (bisbenzimide labeled) per a 50 μm z-stack that included the slices superficial surface.

### Two-photon microscopy

All live-cell imaging was performed with an upright two-photon laser-scanning microscope (Olympus FV1000 MPE, Germany) equipped with an XPLN 25×/1.05 water immersion objective, coupled to a mode-locked Ti:sapphire laser (Mai Tai® DeepSee™, Spectra-Physics, UK) and an incubation chamber surrounding the microscope stage for temperature and CO2 control. Slices on membranes were placed in the (pre-warmed) incubator at least 1 h before imaging and kept at 37°C with 5% CO2 throughout the imaging period (up to 7 days). Tissue was imaged in phenol red free culture media, as this has been suggested to contribute to increased background noise and phototoxicity during long-term imaging (Dailey et al., 2006). Media was perfused into the slice culture dish at a slow rate of approximately 1 ml per hour to avoid the disruption of the slices and subsequent focus artifacts.

When imaging living tissues it is important to keep the exposure to laser light at a minimum to avoid phototoxicity while still maintaining the desired spatial and temporal resolution. This can be done by adjusting the laser power (should be kept at a minimum), exposure time (by adjusting the speed of scanning, or pixel dwell time), the frequency of scans (interval between z-stacks), the size of optical sections (z-stack sampling interval as well as x,y resolution), or the total z-stack thickness. We optimized these parameters for our purposes, however each parameter will depend on the microscope, fluorophore, and specific aim of the study (i.e., whether spatial or temporal resolution is more highly desired), and can be easily adjusted accordingly. In this study, GFP was imaged using an excitation wavelength of 890 nm, at a scan speed of 10 μs/pixel and a resolution of 1024 × 1024. The laser power that slices were exposed to was ≤3 mW after the objective, which is with safe limits to avoid phototoxicity during multi-photon live-cell imaging (König, 2006). Optical sections were collected in z-stacks of 100–150 μm at intervals of 1 μm and complete z-stack scans were carried out every ~30 min. This sampling frequency was adequate for the cell behavior of interest in this study and produced high spatially resolved images for 3-D reconstruction while still allowing individual cells to be tracked over time. One caveat to acquiring large z-stacks with high spatial resolution over extended periods is the manageability of file sizes for data storage as well as processing power for data analysis, including 3D and 4D reconstructions; these considerations should be taken into account when planning experiments as adequate data storage systems, computing power and data analysis software are an absolute necessity. If a higher temporal sampling frequency between z-stacks was needed, total z-stack thickness was decreased and z-stacks were collected every ~10 min without sacrificing spatial resolution or affecting tissue viability. Imaging began at least 50 μm below the surface of the slice to avoid tissue which may be damaged during slicing. At the end of the imaging sessions slices were immediately fixed in 4% PFA for further analysis.

### Immunohistochemistry

After culture and imaging, slices were fixed in 4% PFA at 4°C for 4 h, and washed thoroughly in PBS. Slices were blocked in 10% normal horse serum (NHS) and 0.4% Triton-X in PBS for 1 h and incubated in primary antibody (in PBS, 2.5% NHS and 0.4% Triton-X) for 48 h at 4°C. Primary antibodies included: rabbit anti-Olig2 and mouse anti-NeuN (1:500; Millipore, Germany), rat anti-Ki67 (1:500; eBiosciences, San Diego, CA), goat anti-GFAP (1:2000) and goat anti-SOX2 (1:500; Abcam, UK) and rabbit anti-GFAP (1:500; Dako, Denmark). After PBS rinses, slices were incubated in the appropriate secondary antibody conjugated to Alexafluor 405, 594, or 633 (1:500; Life Technologies, Carlsbad, CA) in PBS with 2.5% NHS and 0.4% Triton-X overnight at 4°C. Slices were washed in PBS, placed between two coverslips with a 400 um thick slice of agarose to act as a spacer if needed and mounted in Fluoromount-G (eBiosciences, San Diego, CA).

All fixed tissue was imaged with a laser-scanning confocal microscope (Olympus FV1000, Germany) equipped with a UV diode (405 nm excitation) and three laser lines (488, 543, 633 nm) along with the appropriate emission filter sets; this configuration allowed for imaging of GFP expression and up to three additional markers in a single slice (for example see Figure 4A).

### Data and image analysis

Two-photon and confocal images are presented as maximum intensity projections of the imaged z-stack. Projections were made using Imaris (Bitplane, Switzerland) or Olympus Fluoview software (FV10-ASW 4.0 Viewer). Adobe Photoshop was used to adjust for brightness and contrast. Image analysis (cell counts, cell migration tracking, 4D analysis, and movies) was done using Imaris. Image J was used to align time points and correct for occasional drift that occurred during imaging using the Linear Stack Alignment with SIFT plugin (Lowe, 2004). Graphpad Prism was used for graphs and statistical analysis; statistical significance from cell counts was determined using Welch's *t*-test. All results are reported as mean ± standard error.

## Results and discussion

### Organotypic tissue slices

Organotypic slice cultures are extremely beneficial when a region of interest is not optically accessible *in vivo*; this *ex vivo* culture method preserves the cytoarchitecture and the microenvironment of the tissue, and allows for cell-cell interactions in three-dimensional space (Gahwiler et al., 1997). In this study we used the interface method (Stoppini et al., 1991), which is most often used with cortical and hippocampal slices, to culture organotypic slices of the developing spinal cord that allowed us to directly observe progenitor cell behavior. The examples presented are of embryos electroporated at E12-E16 and imaged 24–168 h later, however this method can also be used for other embryonic ages as well as early postnatal tissues (e.g., P1–P7). We have found that more immature tissues have better viability in culture and can therefore be imaged over longer intervals, however older tissues tend to maintain greater cytoarchitectural integrity in culture.

### Transfection

Tissue slice electroporation was most efficiently carried out with Genepaddles™ when tissue was embedded in agarose and paddles placed on either side of the agarose rather than directly in contact with tissue (Figure 1A). The agarose additionally acted as a weight to anchor tissue onto the membrane, thus increasing stability during imaging and providing structure surrounding the tissue slice. Approximately 12 h after transfection of the BLBP-GFP plasmid, GFP expression was present at the surface of the tissue to a depth of ~150 μm (for example see Figure 1F; Supplementary Material Movie S1); depth penetration was likely aided by plasmid DNA access into the spinal cord lumen. The BLBP-GFP plasmid has previously been established as radial glial cell specific, largely in the developing cortex in mice (Schmid et al., 2006). We confirmed specificity in the rat spinal cord; GFP expression was found to co-localize in the soma, processes and endfeet of BLBP immunopositive cells (Figure 1B).

Transfection efficiency was estimated as the percentage of SOX2 expressing cells (a specific marker for progenitor cells) that also expressed GFP after 24 h *in vitro* (see Figure 1C). We found that an average of 10.5% ± 0.9 of SOX2 expressing cells also expressed GFP, with a transfection efficiency range of 1.3–38.2% (*n* = 73). The number of labeled cells was variable between cases and was likely dependent on a number of factors including slice health, specific slice region (i.e., along the caudal-rostral axis of the spinal cord) and the age of embryos at the time of transfection [older slices (e.g., E16) had a lower average transfection efficiency at 4.6% ± 0.6, *n* = 22]. However, transfection efficiency was less variable in slices electroporated concurrently and from the same embryo (e.g., six tissue slices harvested from one E14 embryo had an average transfection efficiency of 11.7% ± 1.5, range 7.1–16.8%).

An average transfection efficiency of ~10% was ideal for the direct visualization of progenitor cell behavior; excessive GFP labeling made it optically difficult to separate and track individual cells over long periods of time. It has also been shown that excessive concentrations of fluorophores may lead to an increase in phototoxic effects (Galdeen and North, 2011). For the same reasons, this method has advantages over using slices from transgenic animals where every cell of a specific population is labeled (e.g., transgenic mice expressing GFP in all radial glial cells; Schmid et al., 2006). Transfecting slices directly in this manner has the added benefit of being strain and species independent (i.e., plasmid DNA can easily be transfected into different mouse and rat strains, or into other species) and allows for transfection with transgenes that might normally be embryonically lethal.

### Tissue viability

We assessed cell death in tissue slices using PI, a marker for membrane integrity (Macklis and Madison, 1990). An average of 7.3% (± 3.6, *n* = 6) of cells were PI+ immediately after slicing, indicating the levels of naturally occurring cell death as well as cell damage due to the tissue slicing process (0 DIV; Figure 1D); in these slices the immediate surface of the slice contained many of the PI+ cells, as may be expected due to mechanical damage during slicing. We found no significant difference in the number of PI+ cells between electroporated and non-electroporated slices (Figure 1D); indicating that slice electroporation was not detrimental to tissue viability. Averaging all slices, after 2 DIV we found a small but significant increase in cell death (12.6% ± 6.5, *n* = 13) compared with 0 DIV slices (*p* = 0.0273). This increase was likely due to residual and delayed damage due to the tissue slicing process combined with the process of the tissue slice adjusting to the culture environment. After 4 DIV the percentage of cell death significantly decreased (4.1% ± 2.4, *n* = 9; *p* < 0.0001) and after 6 DIV it decreased further (1.7% ± 0.9, *n* = 11), resulting in significantly fewer PI+ cells, even when compared with 0 DIV slices (*p* = 0.0191). Therefore, there is a period of increased cell death in earlier embryonic ages and in the first few days of culture as the slices recover and adjust to the culture environment, but by 6 DIV cell death in slices has stabilized. That there is some ongoing cell death at this stage is not surprising as during normal development of the spinal cord there is considerable programmed cell death occurring, largely due to the death of excess motorneurons (Oppenheim, 1991; Yamamoto and Henderson, 1999).

### Two-photon time-lapse imaging

Two photon time-lapse imaging was carried out continuously up to 168 h (7 days) post-transfection or until cells showed signs of being unhealthy (e.g., retraction and blebbing of processes, shrinkage, and lysis of cell somata), after which slices where immediately fixed and processed for immunohistochemistry. During imaging, GFP expression was followed both at the single-cell level with a spatial resolution appropriate to visualize fine processes (Figure 1E) and in whole populations of cells (Figure 1F; Supplementary Material Movie S1) in large 3-D blocks of tissue (approximately 500 by 500 by 150 μm).

Previous studies have used other microscopy methods to study the properties of the developing spinal cord; however, usually by necessity, either spatial or temporal resolution has to be compromised. Although, neuroepithelial cell development in spinal cord segments of chick embryos has been captured through 45 μm of tissue using widefield microscopy for optimally 24–48 h (Das et al., 2012), the two-photon method described here allows access to tissue depths up to several hundred microns, thus capturing the behavior of larger cell populations at greater resolution. Higher resolution confocal imaging of rodent progenitor cell behavior in spinal cord slices over time has been carried but only for brief imaging periods (up to a maximum of 13 h), due to the inherent phototoxicity of the confocal laser illumination (O'Leary and McDermott, 2011). To our knowledge the only two-photon imaging of immature spinal cord slices has been undertaken to investigate neuronal firing patterns acutely (5–20 h duration) using calcium indicator dyes in neonatal tissue. (Bonnot et al., 2005; for review see O'Donovan et al., 2008). The advantage of the imaging method described here is that it allows us to leave tissue slices in the imaging chamber for multiple days, enabling continuous, long-term live-cell imaging in a stable tissue culture environment. Additionally, by using two-photon imaging we achieve increased depth penetration (Helmchen and Denk, 2005) and tissue viability (i.e., exposure to lower levels of laser power and phototoxicity), while achieving efficient fluorescence collection (in comparison to confocal imaging involving a pinhole) and maintaining high spatial and temporal resolution for the detailed observation of developmentally regulated progenitor cell behavior. Considering the substantial advantages offered by two-photon imaging for *in vivo* investigations (Stosiek et al., 2003; Garaschuk et al., 2006) it is not surprising that using two-photon imaging can offer similar advantages for imaging living tissues over extended time periods in an *ex vivo* model—with the added benefit of considerably greater flexibility in relation to the control and manipulation of environmental parameters.

### Proliferation

**Figure 2:**
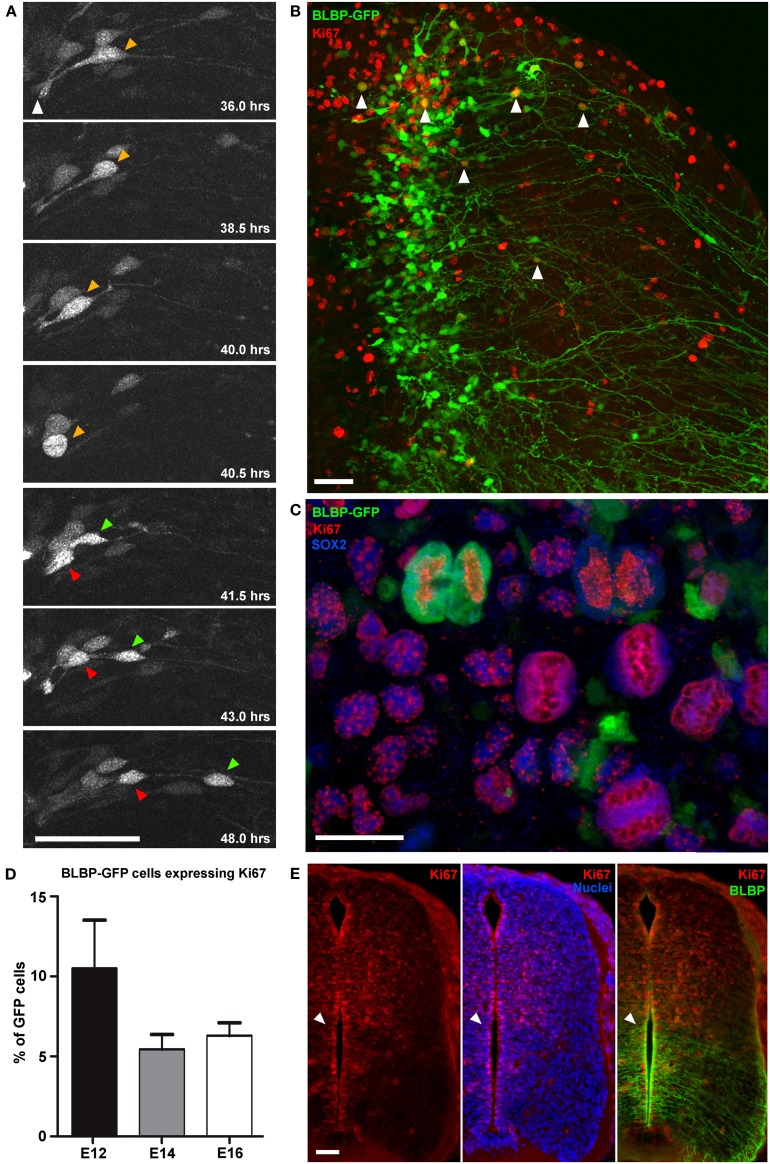
**Direct observation of cell division in BLBP-GFP transfected cells. (A)** Two-photon image sequence of a GFP-expressing cell in a spinal cord slice undergoing cell division at ~40 h after BLBP-GFP transfection at E12. Orange arrow indicates cell before division with apical attachment to the lumen (white arrow). Before division the cell exhibits interkinetic motion toward the ventricular surface and rounds up (40.5 h timepoint); after division one daughter cell begins to migrate out toward the pial surface (green arrow) and one cell (red arrow) maintains the apical attachment to the lumen and undergoes interkinetic motion again to return to a similar position in the ventricular zone as before division. **(B)** Confocal image of a spinal cord slice electroporated at E14 and fixed after 2DIV showing BLBP-GFP expression (green) and Ki67 immuno labeling (red); only a small number of cells (white arrowheads) are colabelled. **(C)** Confocal image of a single late telophase GFP+(green), SOX2+(blue), Ki67+(red) cell and several Ki67+SOX2+ cells, indicating preserved proliferative capacity and slice viability. **(D)** Quantification of BLBP-GFP cell proliferation in tissue slices electroporated at E12, E14, and E16 and cultured for up to 7 days. There were no significant differences in the levels of proliferation between different embryonic ages. **(E)** Immunofluorescently stained 20 μm cryostat section of a fixed E14 embryo comparing Ki67 staining (red channel only—right panel), Ki67 and bisbenzimide staining (red and blue channel—middle panel) and Ki67 and BLBP staining(red and green channel—left panel), indicating the increased levels of BLBP expression ventrally where there are fewer actively proliferating cells (Ki67+). Bisbenzimide nuclear staining (blue) demonstrates that the lack of Ki67+ cells ventrally is not due to the paucity of cells in this region. White arrowhead indicate the transition between high BLBP-expression ventrally vs. high numbers of Ki67+ cells dorsally. In all photomicrographs dorsal is oriented toward the top, ventricular zone toward the left and the pial surface is toward the right of the image. Scale bars in **(A,B,E)** = 50 μm; **(C)** = 25 μm.

Using two-photon imaging, dividing GFP-expressing cells were recorded with high resolution (Figure 2A). Generally, dividing cells had apical attachments to the ventricular surface. The cell soma went through interkinetic motion until it reached the apical attachment and then rounded up (see 40.5 h timepoint in Figure 2A) before dividing. However, relatively few GFP-expressing cells were seen undergoing divisions in the time-lapse imaging. For example, the dividing cell in Figure 2A was one of only four cells out of 78 imaged cells that were seen dividing over a 30 h imaging period (from 24–50 h *in vitro*).

This low level of proliferation of GFP-expressing cells in slices was further confirmed using Ki67 immunohistochemistry and confocal imaging after time in culture, both at the level of the whole population (Figure 2B) and in immunohistochemically phenotyped cells (Figures 2B,C). In cultured slices, we found that on average fewer than 10% of GFP-expressing cells were also Ki67 immunopositive (7.3% ± 1.1, *n* = 67; Figure 2D). Nonetheless, there were considerable numbers of Ki67 immunopositive cells that were non-GFP expressing, further indicating the growth potential and viability of the slice preparation. BLBP expression in the spinal cord is known to follow a ventral to dorsal developmental gradient first appearing ventrally around E12\13 in the rat spinal cord. With increasing developmental age BLBP then becomes expressed in radial glial cells in dorsal regions of the spinal cord (Barry and McDermott, 2005). In cyrosections of comparable ages we found that regions of the spinal cord which contain most BLBP + radial glial cells have substantially less Ki67 staining. In fact, a strikingly abrupt transition between regions of high BLBP expression ventrally and high Ki67 expression dorsally is evident (Figure 2E), confirming and explaining the very low level of division measured in BLBP-GFP + radial glial in slices. This suggests that, at least in the spinal cord, once neuroepithelial cells transition into radial glial cells (as defined by the expression of BLBP), the amount of cell proliferation decreases, and less than 10% of this progenitor pool continues to divide.

In the cerebral cortex, radial glial cells (and related neuroepithelial cells) have been shown to undergo symmetric or asymmetric divisions, generating a combination of either daughter radial glial cells, intermediate progenitor cells, or neurons directly (Weissman et al., 2003; Attardo et al., 2008; Noctor et al., 2008; Kriegstein and Alvarez-Buylla, 2009; Asami et al., 2011; Shitamukai and Matsuzaki, 2012; Pilz et al., 2013). Because of the very small number of actively proliferating BLBP-GFP labeled cells that we found in the spinal cord, it is difficult to classify the division events in the same way that has been described in the developing cortex. Many questions remain regarding CNS progenitor cell division in general, and specifically, the progenitor potential of radial glial cells in the spinal cord and their contribution to neurogenesis. The method described here enables detailed qualitative as well as quantitative investigations of cell division events in the developing spinal cord. Additionally, it allows for investigations into micro-environmental factors affecting cell proliferation in live tissue slices.

### Migration

**Figure 3:**
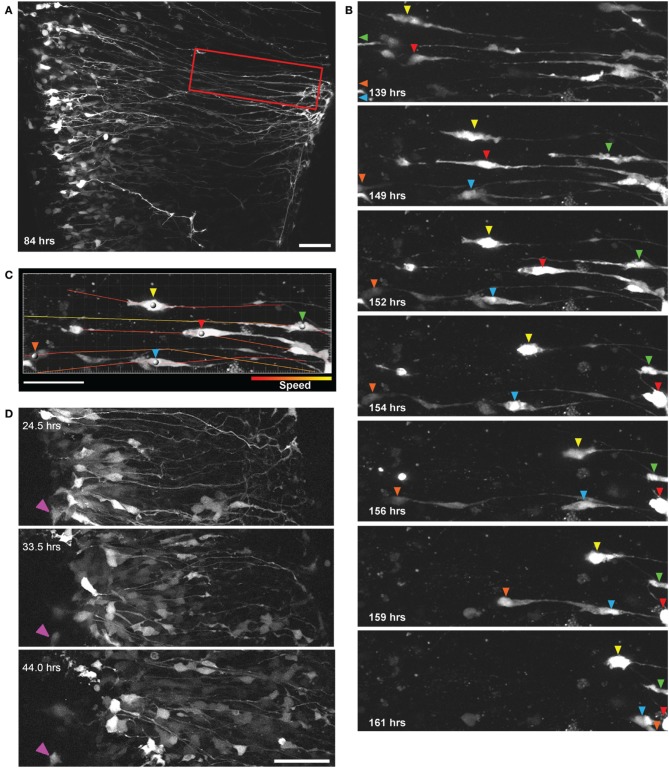
**Direct observation of radial glial cell migration using two-photon extended time-lapse imaging. (A)** GFP expression in a population of radial glial cells 84 h after transfection with BLBP-GFP plasmid at E14; cell somata are aligned in the ventricular zone with radial processes extending out toward the pial surface. Region outlined in red is shown in **(B)** where individual GFP labeled cells (differentiated by colored arrows) can be seen migrating out to the pial surface using somal translocation over a time period of 22 h beginning 139 h after transfection. **(C)** Parameters such as migration speed can be analyzed using Imaris software; here the migratory tracks taken by the individual cell soma [colored arrows in **(B)**] are shown overlaid on a single frame and color-coded according to the speed of migration. **(D)** Two-photon imaging of many GFP labeled cells migrating over a 20 h period away from the ventricular zone in a spinal cord slice electroporated at E12; purple arrow indicates a stationary cell and is shown for a reference point across frames. In all photomicrographs dorsal is oriented toward the top, ventricular zone toward the left and the pial surface is toward the right of the image. All scalebars = 50 μm.

Previous research in the spinal cord, using fixed tissue sections, suggests that in later developmental stages when radial glia terminally differentiate into astrocytes they migrate to the pial surface (Barry and McDermott, 2005), however, this has not been shown directly in living cells and the mechanisms of migration have not been demonstrated in the spinal cord. Using the methods described we recorded significant radial migration of GFP-expressing cells out toward the pial surface. We also found that this migration proceeds largely through somal translocation, where cells have a leading process with pial attachments and by shortening this process the cell soma progressively travels from the ventricular zone (VZ) out toward the pial surface. Figure 3A shows a two-photon image of GFP-expressing radial glial cells in a spinal cord slice 84 h after electroporation at E14; radial glia cell bodies are located in the VZ and radial processes extended out to the pial surface. In Figure 3B a subset of these cells are highlighted, clearly migrating from the VZ to the pial surface between 139 and 161 h (see also Supplementary Material Movie S2).

This method allows several migration parameters to be studied, including migration speed (Figure 3C), displacement, direction and mode of migration, as well as detailed cell tracking in large, identified cell populations in organotypically organized living tissue blocks (Figure 3D). It has been shown in acute brain slices of the developing cerebral cortex that cortical neurons migrate radially from proliferative zones out to their destination through two distinct modes, somal translocation and/or glial-guided locomotion (Nadarajah et al., 2001, 2003; Ayala et al., 2007). Despite these important studies, regional differences in the modes and mechanisms of migratory movements that are used throughout the CNS, are not fully understood. In mammals, the migratory strategies of cells developing in the spinal cord when compared with the layered and expansive cerebral cortex are quite different (Rakic, 2003), as is the specific timeline during development (Gotz and Huttner, 2005); it stands to reason that the modes and mechanisms of cell migration may also be quite disparate between these CNS regions. Previous research investigating cell migration in the spinal cord has been limited, either due to the lack of live imaging (Leber and Sanes, 1995; Phelps et al., 1996) or too brief an imaging duration (Tsai et al., 2009; O'Leary and McDermott, 2011), which may not be sufficient to capture relevant migratory events of whole populations (for example in Figure 3B the majority of migratory events occur only after 4 DIV); the method described here would be useful in addressing many remaining questions in the spinal cord as well as other CNS regions.

### Differentiation

**Figure 4:**
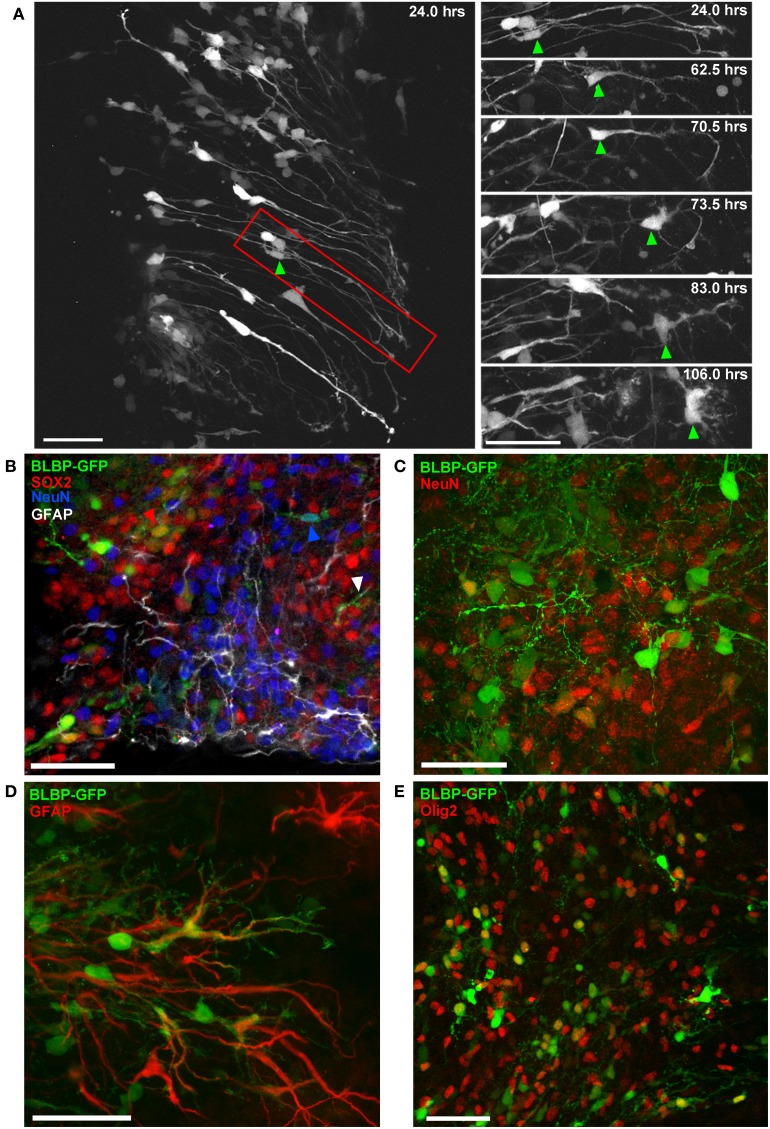
**Differentiation of BLBP-GFP expressing cells in spinal cord organotypic slice cultures. (A)** Two-photon time-lapse sequence showing population of GFP-expressing cells (left panel) 24 h after electroporation at E12. Region outlined in red is shown in selected time-lapse frames on the right. The cell indicated by the green arrow initially displays a radial morphology. Morphological changes occur over an 82 h time period, after which the cell has acquired a multipolar morphology. **(B–E)** Using confocal microscopy and immunohistochemistry the contribution of BLBP-expressing cells to the generation of different neural cell lineages can be examined in detail **(B)** Triple immuno-phenotyping of GFP-expressing cells (green) transfected at E12 and fixed after 7 DIV demonstrates the generation of progenitor cells (SOX2+:red arrow), cells of neuronal (NeuN+: blue arrow), and astroglial lineage (GFAP+: white arrow). **(C,D)** show further examples of GFP-expressing cells co-expressing NeuN and GFAP, respectively. **(E)** The co-expression of GFP and Olig2, an oligodendrocyte marker, is also evident. In all photomicrographs dorsal is oriented toward the top, ventricular zone toward the left top and the pial surface is toward the right of the image. Scalebars in **(A,B,D)** = 50 μm; **(C,E)** = 25 μm.

This method also allows us to record detailed morphological changes in individual cells over extended time periods. Figure 4A shows a two-photon image sequence of a single cell (right panel) isolated from a population (left panel) of radial glial cells transfected at E12; gradual morphological development from a radial to a multipolar cell is evident over a period of 5 DIV. Following imaging, transfected cells were phenotyped using slice immunohistochemistry and confocal imaging for up to three phenotypic markers simultaneously (e.g., Figure 4B); in this way, we can determine the potential for labeled progenitor cells to generate neurons (Figure 4C), glia (Figures 4D,E) or remain in an undifferentiated state (SOX2; Figure 4B).

Previous studies using lineage tracing techniques have demonstrated that “radial cells” in the cerebral cortex contribute significantly to neurogenesis as well as gliogenesis (Noctor et al., 2001; Anthony et al., 2004; Haubensak et al., 2004; Pinto et al., 2008; Betizeau et al., 2013). However, lineage tracing studies are often limited in that GFP expression is not always cell-type specific and cell phenotyping is often based purely on morphology. Hence it is not always clear to what lineage, neuroepithelial cell or specifically radial glial cell, the findings apply; in fact the definition of these two cell types has indeed become unclear (for a good description see Gotz and Barde, 2005). While these studies have greatly contributed to our understanding of progenitor cell function in the developing cerebral cortex, our grasp of the diverse nature and cytogenic potential of radial glial cells in all CNS regions continues to expand (Kriegstein and Gotz, 2003; Malatesta et al., 2003; Anthony et al., 2004; Pinto and Gotz, 2007; Costa et al., 2010; Wang et al., 2011; Pilz et al., 2013). Many questions remain regarding the progenitor potential of radial glial cells, particularly in the spinal cord. This *ex vivo* embryonic spinal cord model provides a unique system where radial glial cell differentiation can be directly observed as well as experimentally manipulated.

### Conclusion

We have established a novel time-lapse imaging protocol using two-photon microscopy to directly observe progenitor cell behavior in spinal cord slices over extended periods of time during development. This approach allows continuous imaging of living tissue for up to 7 days in culture with high spatial and temporal resolution. The use of slice electroporation allows us to genetically target specific cell populations (e.g., BLBP expressing radial glial cells). The use of two-photon imaging reduces phototoxic effects while allowing high resolution imaging of large regions of tissue in the x, y and z dimension, and is particularly important for efficient imaging deep within tissue. The major advantage of this approach is the potential for detailed examination of cell behavior both at the population level and the level of the individual cell. Although we have presented this approach as a method for imaging radial glial cells in the developing spinal cord, this protocol can be applied to the study of various cell types in other regions of the embryonic CNS, as well as other developing tissue types that have been shown to be viable in culture (for example cerebral organoids, Bershteyn and Kriegstein, 2013; Lancaster et al., 2013).

### Conflict of interest statement

The authors declare that the research was conducted in the absence of any commercial or financial relationships that could be construed as a potential conflict of interest.

## Supplementary material

Supplementary Material Movie S1 **3D high resolution two-photon imaging of GFP-expressing cells in a developing spinal cord slice**. Two-photon z-stack at a single time point is shown with GFP-expressing radial glial cells 84 h after electroporation at E14. The 3D imaging area of the tissue block is approximately 500 × 500 × 150 μm with high spatial resolution, even along the z-axis, as is evident when the z-stack is divided by a clipping plane (yellow frame) showing clearly identifiable individual fibers throughout the tissue depth. In the opening frame dorsal is toward the top and the pial surface is toward the right of the frame. Single grid square is 50 × 50 μm.

Supplementary Material Movie S2 **4D two-photon time-lapse imaging enables direct observation of radial glial cell migration**. GFP-expressing cells are shown after transfection with BLBP-GFP plasmid at E14. A single timepoint (125 h after electroporation) is shown initially with high spatial resolution. As the time-lapse imaging proceeds (one complete z-stack acquired every ~30 min), GFP-expressing cells can be seen migrating out toward the pial surface using somal translocation over a time period of 10 h (out of the 168 h total imaging time) at a high spatial and temporal resolution. In this sequence dorsal is toward the top and the pial surface is toward the right of the frame. Single grid square is 20 × 20 μm.
